# Supplementary material for: RNA-Seq Virus Fraction in Lake Baikal and Treated Wastewaters
Source: Int J Mol Sci. 2023 Jul 27;24(15):12049. doi: 10.3390/ijms241512049 (PMC10418309; doi:10.3390/ijms241512049)
Supplement: Supplementary file 1 [file ijms-24-12049-s001.zip › Figure S1.pdf]

A

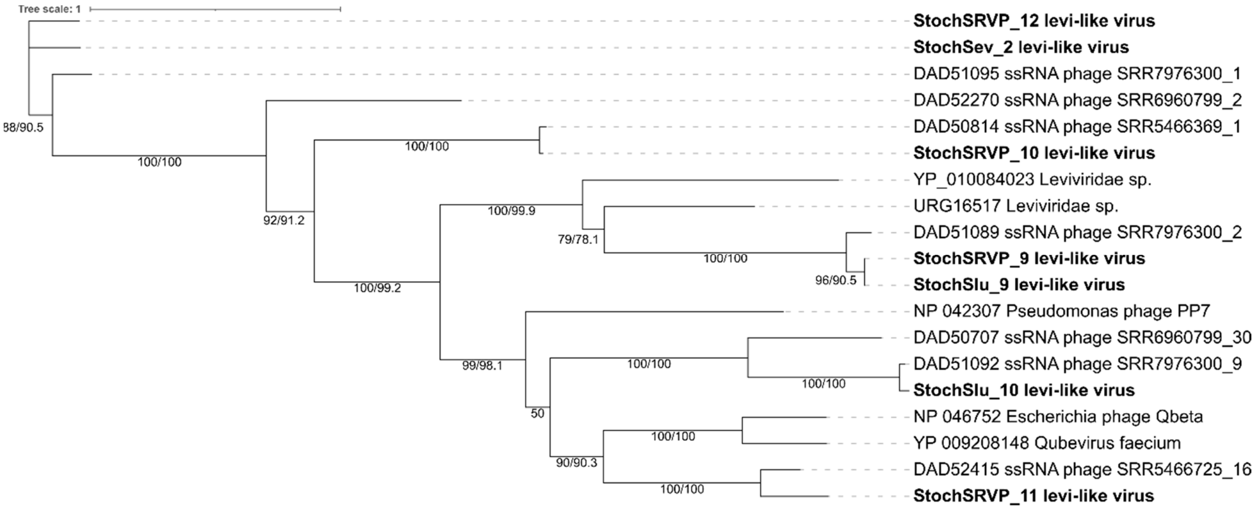

Best-fit model according to BIC: VT+F+R3

B

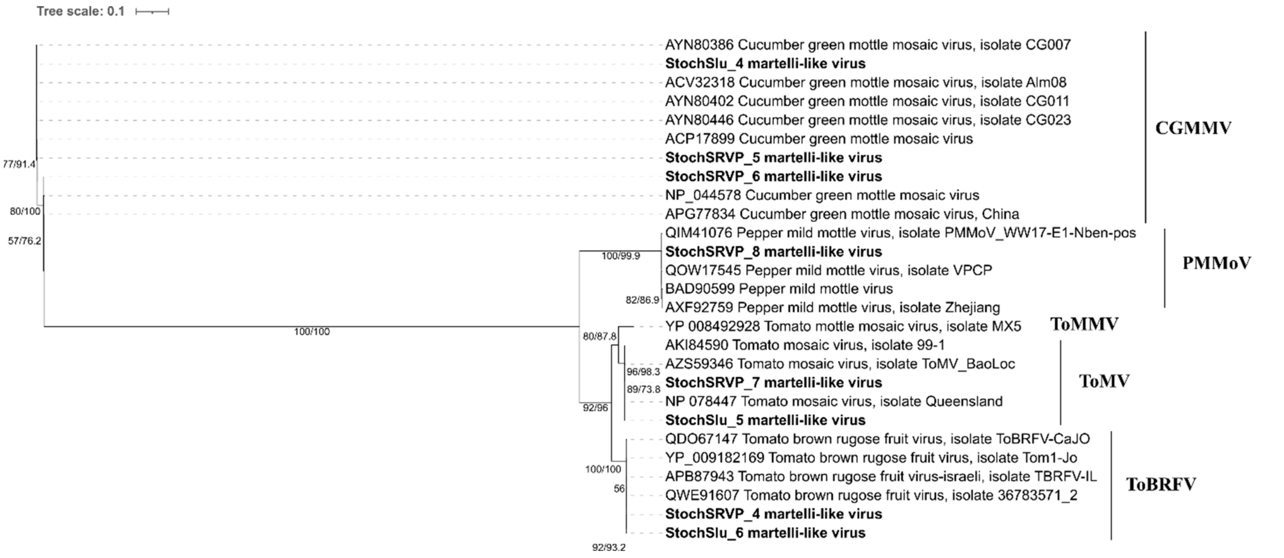

Best-fit model according to BIC: LG+G4

C

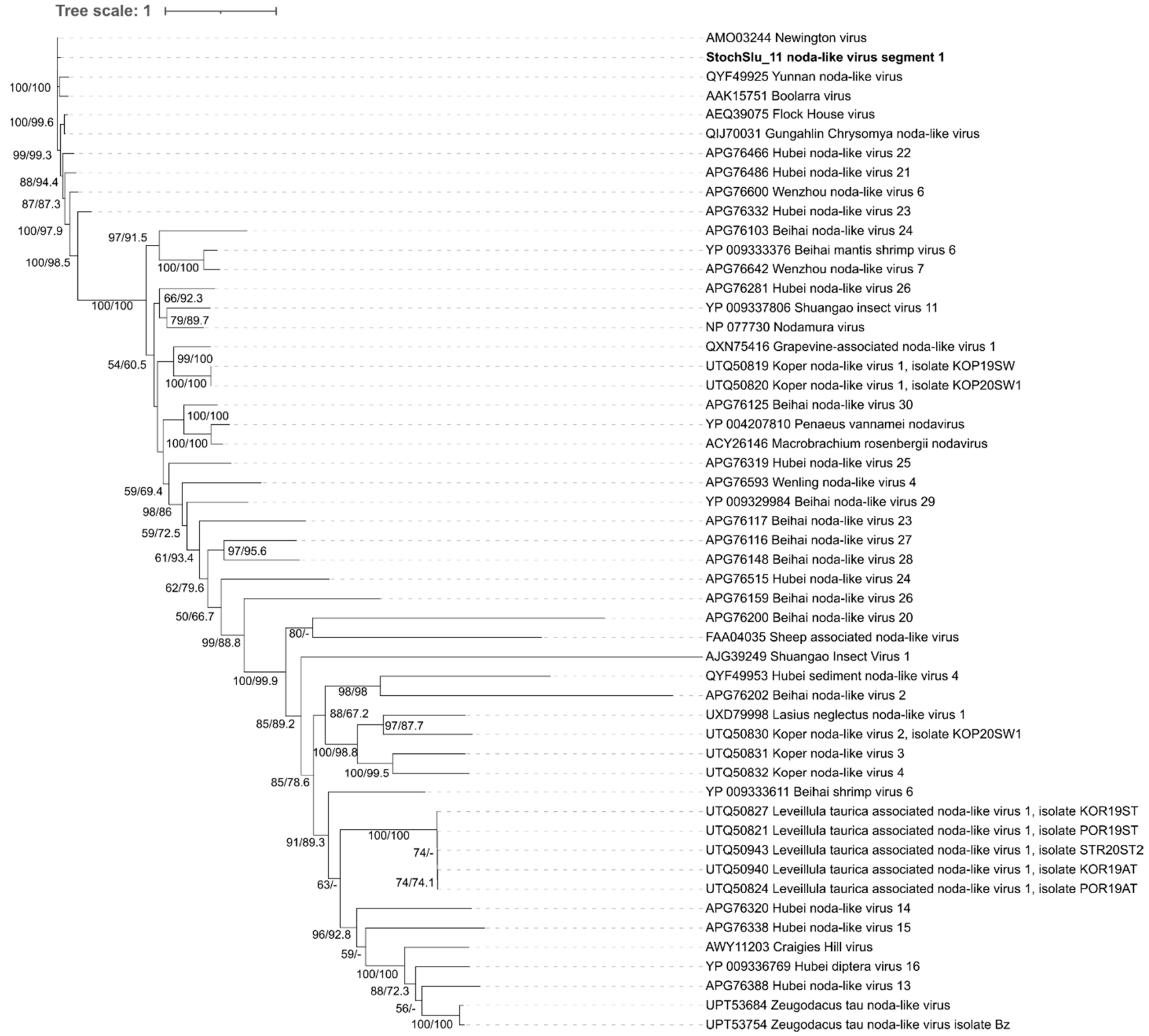

Best-fit model according to BIC: LG+R5

D

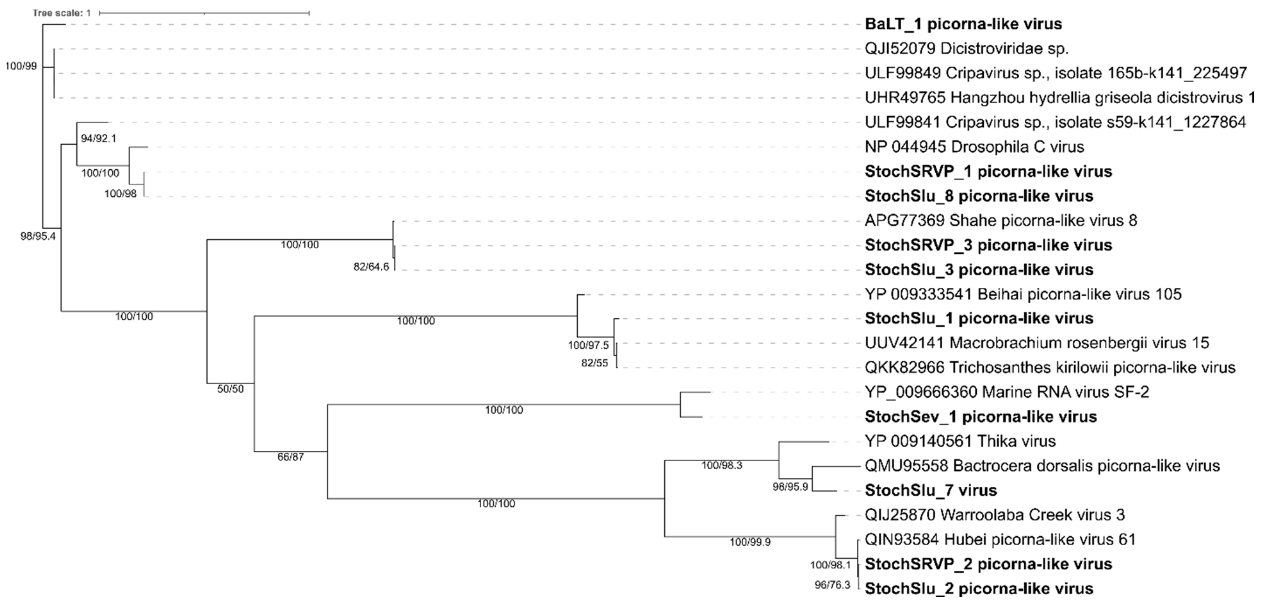

Best-fit model according to BIC: LG+I+G4

Figure S1. Maximum likelihood phylogenetic trees constructed using IQ-TREE based on amino acid RdRp sequences from the genomes of this study and sequences from GenBank. Branch support was calculated using the the UFBoot (ultrafast bootstrap) algorithm and approximate Likelihood Ratio Test (SH-aLRT) on 1000 replications. A - levi-like viruses, B - martelli-like viruses, C - noda-like viruses, D - picorna-like viruses. Sequences obtained in this study are shown in bold. Bootstrap values greater than 50% are shown at branches.
