# Supplementary material for: RNA-Seq Virus Fraction in Lake Baikal and Treated Wastewaters
Source: Int J Mol Sci. 2023 Jul 27;24(15):12049. doi: 10.3390/ijms241512049 (PMC10418309; doi:10.3390/ijms241512049)
Supplement: Supplementary file 1 [file ijms-24-12049-s001.zip › Figure S2.pdf]

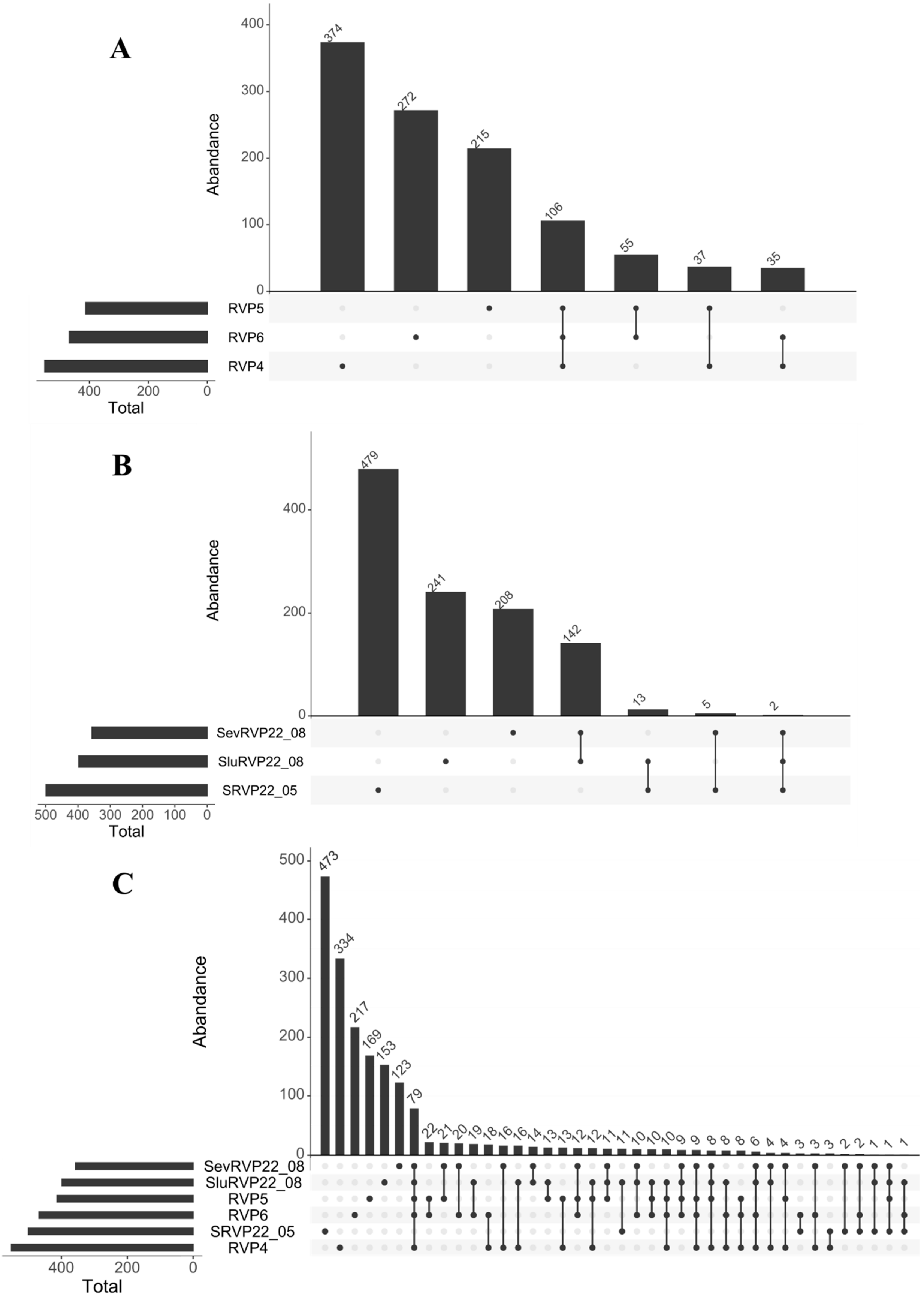

Figure S2. UpSet plot showing the common and unique tax id of UVIG DNA-containing virus proteins from transcriptomes determined using IMG/VR. A - pelagic samples only, B - treated wastewater samples only, C - samples from pelagic and treated wastewater. Dots show unique

tax id present only in this sample, lines show common.
